# Berberine Influences Blood Glucose via Modulating the Gut Microbiome in Grass Carp

**DOI:** 10.3389/fmicb.2019.01066

**Published:** 2019-05-09

**Authors:** Houjun Pan, Zhifei Li, Jun Xie, Duan Liu, Hongjuan Wang, Deguang Yu, Qing Zhang, Zhiyi Hu, Cunbin Shi

**Affiliations:** ^1^Key Laboratory of Tropical and Subtropical Fishery Resources Application and Cultivation, Pearl River Fisheries Research Institute, Chinese Academy of Fishery Sciences, Guangzhou, China; ^2^Business School of Hunan University, Changsha, China; ^3^Health Time Gene Institute, Shenzhen, China; ^4^State Key Laboratory of Biocontrol, School of Life Sciences, Sun Yat-sen University, Guangzhou, China; ^5^State Key Academic Discipline of Guangzhou University of Chinese Medicine, Guangzhou, China

**Keywords:** berberine, gut microbiome, blood glucose, lipids, freshwater fish

## Abstract

Berberine (BBR), an isoquinoline alkaloid, is a major pharmacological component of the Chinese herb *Coptis chinensis*, which has been listed in the Chinese Fisheries Pharmacopeia as a common drug for the control of bacterial fish diseases. However, BBR is poorly absorbed into the systemic circulation but is significantly accumulated in the intestine. It is difficult to explain the mechanism of clinical effects of BBR based on systemic genes and pathways; it has been proved that the function of BBR in mammals is associated with the host metabolic phenotypes mediated by the structural modulation of gut microbiota. The mechanism of pharmacological effects of BBR in fish remains unclear. Here, we fed grass carp (*Ctenopharyngodon idellus*) a diet supplemented with BBR at a dose of 30 mg/Kg body weight daily and compared them with grass carp fed a regular fish feed diet. Biochemical analysis revealed that fish fed BBR had significantly reduced serum glucose, total cholesterol (TC), and triglyceride (TG) levels, and increased TC (*p* < 0.05) and TG (*p* < 0.01) levels in the liver. Deep amplicon sequencing of the V4 region of 16S rRNA genes of the gut microbiota revealed: (i) the composition of gut microbiota after BBR feeding was more diverse than that in the control group; (ii)before fish were fed BBR, the enriched operational taxonomic units (OTUs) mainly belonged to Firmicutes while most enriched OTUs came from Proteobacteria, Planctomycetes, Bacteroidetes, and Firmicutes during BBR feeding and after BBR feeding stopped; (iii) the ratio of Firmicutes to Bacteroidetes was significantly decreased in fish fed BBR. Spearman’s rank correlation showed that 32 berberine-OTUs were significantly negative correlated with glucose (*p* < 0.05). It indicates that BBR may affect the levels of serum glucose by the structural modulation of gut microbiota. Our results provide insight into the effect of BBR on fish metabolism and gut microbiomes, which would be beneficial for the fish welfare.

## Introduction

The grass carp is a herbivorous freshwater fish and one of the most important economic farmed fish in China; it has been introduced into more than 100 countries (Wu et al., 2012; Hao et al., 2017). Generally, fish do not utilize dietary carbohydrate effectively (Furuichi and Yone, 1980; Walton and Cowey, 1982; Shiau, 1997; Stone, 2003; Polakof et al., 2012) and most carnivorous fish exhibit a persistent postprandial hyperglycemia (Furuichi and Yone, 1981; Wilson and Poe, 1987; Harmon et al., 1991; Shiau, 1997; Barma et al., 2006; Gleeson et al., 2007; Polakof et al., 2012). Although grass carp can utilize dietary carbohydrate better than other carnivorous fish species (Stone, 2003), they utilize carbohydrates less efficiently than lipids (Gao et al., 2010). In addition, current commercial feeds have a high carbohydrate content. Thus, commercial feeds have been blamed for causing grass carp diseases, such as “big belly grass carp” (Tian et al., 2004).

Berberine (BBR), an isoquinoline alkaloid, is a major pharmacological component of the Chinese herb *Coptis chinensis* (Huang-Lian, a common herb in traditional Chinese medicine). As a botanical drug, BBR or BBR-containing herbs have been used to treat intestinal infections, particularly bacterial diarrhea, for at least 2000 years in China (Tang et al., 2009). BBR has been listed in the Chinese fisheries pharmacopeia as a prescription drug for the control of bacterial fish diseases (Yang, 2005). Recently, accumulative evidence demonstrated that berberine is clinically effective in anti-diabetes in mammals due to its significant hypoglycemic (glucose-lowering) and hypolipidemic (lipid-lowering) effects (Chen and Xie, 1986; Zhang et al., 2008; Zhang H. et al., 2010; Cicero and Tartagni, 2012; Dong et al., 2013; Lan et al., 2015; Zhao et al., 2017). BBR also show hypolipidemic (lipid-lowering) effects in the freshwater fish, blunt-snout bream (*Megalobrama amblycephala*) (Xu et al., 2017). However, the hypoglycemic (glucose-lowering) effects of BBR in fish have not been reported to date.

The proposed mechanisms of action of BBR include stimulation of glycolysis in peripheral tissue cells (Yin et al., 2008), inhibition of liver gluconeogenesis (Xia et al., 2011), activation of AMP-activated protein kinase in both adipose and muscle tissues (Lee et al., 2006), and upregulated expression of genes involved in lipid metabolism (Lu et al., 2016). A paradox remains regarding the mode of action of BBR due to its poor bioavailability (Zhang et al., 2012; Sun et al., 2016). Pharmacokinetic studies have shown that BBR was poorly absorbed into the body; therefore, the levels of berberine in the blood and target tissues were far below the effective concentrations (Gu et al., 2015). Moreover, it has an extreme low absolute bioavailability of 0.68% in rats (Chen et al., 2011) and the maximum concentration (Cmax) of BBR in the plasma of rats is 4 ng/ml after oral administration of 100 mg/kg BBR (Liu et al., 2009; Zhang et al., 2012). The maximum concentration (Cmax) of BBR in the plasma of tilapia (*Oreochromis niloticus*) is 2.95 ng/mL after oral administration of 30 mg/kg BBR (Qin, 2014). As BBR is poorly absorbed into the systemic circulation but significantly accumulated in the intestine (Gu et al., 2015), the primary action site of BBR is the gut (Sun et al., 2016). It has been proved that the action of BBR in rats is associated with the host metabolic phenotypes mediated by the structural modulation of gut microbiota (Zhang et al., 2012; Zhang et al., 2015b; Li et al., 2016; Wang Y. et al., 2017). Furthermore, BBR directly impacts the gut microbiota, thereby altering bile acid metabolism and activating intestinal farnesoid X receptor, which lead to lipid-lowering effects in mice (Sun et al., 2016; Tian et al., 2018). However, the association between the hypoglycemic/hypolipidemic actions of BBR and the gut microbiome in fish remains unknown.

We conducted a BBR feeding experiment to understand the effects on the community structure of gut microbiome and their association with the levels of glucose and lipids in grass carp. We orally administered 30 mg/Kg fish body weight daily, which is the Highest Permission Dosage for controlling bacterial diseases such as enteritis in fish (Yang, 2005), and measured the glucose, TG and TC levels in serum and liver. We also conducted amplicon sequencing of the prokaryotic 16S rRNA gene V4 region. The results of this study may suggest methods of disease control in farmed freshwater fish using BBR.

## Materials and Methods

### Drug and Diet

Berberine chloride (BBR, analytical reagent) was purchased from Sigma-Aldrich (United States). BBR was suspended in distilled water before being sprayed evenly onto grass carp feeds. The formulated grass carp diet included the following: fish meal, 5 g⋅kg^-1^; soybean meal, 215 g⋅kg^-1^; cottonseed meal, 80 g⋅kg^-1^; rapeseed meal, 200 g⋅kg^-1^; wheat flour, 180 g⋅kg^-1^; rice bran, 150 g⋅kg^-1^; lees powder, 50 g⋅kg^-1^; malt root, 50 g⋅kg^-1^; choline chloride, 20 g⋅kg^-1^; mineral mixture 20 g⋅kg^-1^; vitamin mixture, 30 g⋅Kg^-1^ (Yu et al., 2017).

### Fish and Feed

Juvenile grass carp with mean body weight 34.0 g (standard deviation (SD) 0.73) and mean total length 14.8 cm (SD 0.26) were collected from the same spawn and kindly provided by the farm of PRFRI. The fish were maintained in tanks with filtered water in a flow-through system. All experimental protocols were approved by the animal Ethics Committee of the Guangdong Provincial Zoological Society, China (permit number: GSZ-AW003). To remove parasites or pathogens from the fish and feed, fish skin and gills were checked using a microscope, and bacterial and viral contamination was checked using bacterial isolation and reverse transcription quantitative real-time PCR (RT-PCR), respectively (Zhang L. et al., 2010). The fish were fed 2% of their body weight twice daily for 2 weeks in acclimatization culture prior to the experiment (Wang J. et al., 2017). The experiment used a complete block design (2 treatments: BBR-supplemented and control). For the BBR group, fish were fed with feeds that were supplemented with 30 mg/Kg body weight of BBR daily (Yang, 2005) for the first 7 days. Then, from the 8th to 56th day, BBR fish were fed feeds without the BBR supplement. The fish from the control group were fed with feeds without BBR throughout the whole experimental period. Water temperature, dissolved oxygen, ammonia-nitrogen, and nitrite nitrogen were maintained at approximately 28 ± 1°C, 5–6 mg⋅L^-1^, and <0.35 mg⋅L^-1^, and <0.01 mg⋅L^-1^, respectively. For 56 days, the fish received a daily feeding rate of approximately 2% body weight. The weight of fish in each group was determined 0, 7, 14, 28, 42, and 56 and the daily ration adjusted accordingly.

### Growth Performance and Sample Collection

Before sampling collection, fish were anesthetized by immersing in 60 mg⋅L^-1^ MS-222 (Sigma, United States). Then the body length and weight of each fish was measured. Random samples of blood, liver, and the hindgut were taken from the BBR group and control group on days 0, 1, 3, 7, 9, 14, 21, and 28 under strictly sterile conditions. Fish bled from the caudal vein using non-heparinized sterile syringes. To isolate serum, blood samples were left at room temperature in sterile centrifuge tubes for 30 min to allow clotting, and then centrifuged at 3000 ×*g* for 10 min at 4°C (Xu et al., 2017) to collect the supernatant. Serum samples were stored at -80°C for further biochemical analyses. For liver tissues and hindgut samples, grass carp were dissected on a sterile bench. Approximately 0.5–1.0 g of liver tissue was collected. Approximated 2.0 cm of hindgut containing the contents of the intestinal tract was collected from each fish. Each sample (liver or hindgut) was pooled into a 2.0-mL pre-labeled aseptic Eppendorf tube, and then immediately placed into liquid nitrogen and stored at -80°C for further analysis (Lu et al., 2016). All samples collected under strictly sterile conditions. For each sample collection, we collected 10 fish each timepoint from the BBR and control groups.

### Biochemical Assays of Serum and Liver

The levels of glucose, total cholesterol (TC), and triglyceride (TG) in the serum, and TC and TG in the liver were measured using commercial kits (Kehua, China) and a fully automatic biochemical analyzer (Hitachi 7020, Japan) following the manufacturer instructions. Briefly, the glucose level was measured using the GOD-PAP method (Zhang et al., 2012). TC and TG levels were measured using the colorimetric enzyme COD-PAP and GPO-PAP methods, respectively (Xu et al., 2017). SPSS software (version 20.0, IBM, United States) was used to analyze biochemical data. The differences between the BBR and control groups were assessed using a One-way ANOVA test. Statistical significance was determined at *p* < 0.05.

### DNA Extraction, Amplification, and Sequencing of 16S rRNA Genes

All genomic DNA of fish gut microbes was extracted using an E.Z.N.A.^®^ stool DNA Kit (Omega, United States) following the manufacturer instructions under required aseptic conditions (Li et al., 2017). The quality, integrity, and concentration of each DNA sample was determined by 1% agarose gel electrophoresis and a NanoDrop ND-2000 spectrophotometer (Thermo Fisher Scientific, United States). We used the primer set (515F and 806R) and methods previously described (Caporaso et al., 2011, 2012) for PCR amplification of the V4 hypervariable region of 16S rRNA genes (Liu et al., 2016). The pair-end library construction and sequencing of 16S rDNA amplicons was carried out using an Illumina HiSeq 2500 sequencing platform. All raw sequences were deposited in the NCBI Sequence Read Archive with SRA number SRP142659.

### Sequence Analysis

Raw sequences were de-multiplexed, trimmed, and filtered to remove low-quality reads using the open-source software system Quantitative Insights into Microbial Ecology (QIIME) quality filters (Caporaso et al., 2010). The high-quality, paired-end reads were merged to generate the 16S rDNA V4 fragment sequences using FLASH software (Magoc and Salzberg, 2011). Then, all the merged sequences were mixed to pick Operational Taxonomic Units (OTUs) with an identity threshold of 97% using the UPARSE pipeline (Edgar, 2013). The representative sequences for each OTU, RDP classifier tools and the Silva database were used to obtain the taxonomic information for the OTUs. OTUs that were defined as “Unknown,” “Cyanobacteria,” “chloroplast,” or “mitochondria” were removed. The normalized OTU abundance profile was generated for downstream analysis on the assumption that the raw OTU read counts were rarefied to the same counts for each sample.

### Diversity and Statistical Analysis

Based on the normalized OTU abundance profile, the four alpha diversity indices (Chao1, Shannon, Observed species, and Phylogenetic distance whole tree) were calculated to estimate the species diversity and richness for each sample using QIIME software (Caporaso et al., 2010). The rarefaction curves of the four alpha diversity indices were obtained using a maximum rarefaction depth of 30,000 reads. The distances of fish gut microbial communities between different samples were calculated using Bray-Curtis, weighted and unweighted UniFrac beta-diversity metrics. Welch’s *t*-test for two sample groups and Kruskal-Wallis rank sum test for multiple sample groups was used to identify the significant differences in alpha and beta diversity between and among different groups (Zhang et al., 2013). We also used the Kruskal-Wallis rank sum test for multiple sample groups and Wilcoxon rank sum test for two sample groups to identify the significantly enriched OTUs for each treatment group (Zhang et al., 2013). The *P*-values were corrected using the Benjamini and Hochberg (1995) method to account for multiple statistical testing. Correlations between the phenotypic traits and significantly enriched OTUs were calculated using Spearman’s rank correlation (Zhang et al., 2015a). Significant correlations are shown using Heatmap in R software.

## Results

### Berberine Significantly Affected the Levels of Glucose and Lipids in Grass Carp

**FIGURE 1:**
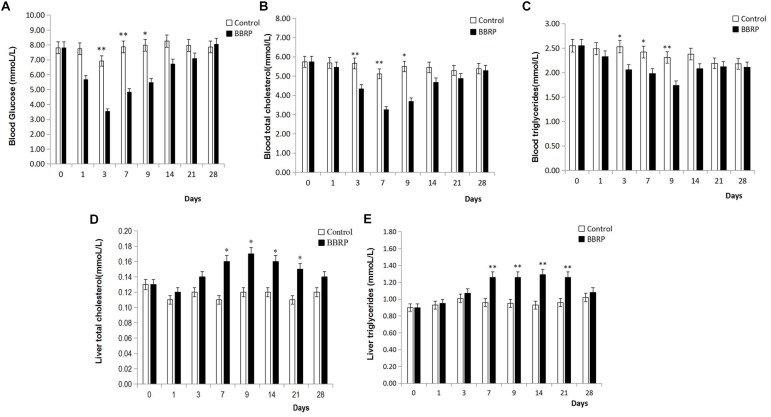
Effect of berberine (BBR) on serum and liver biochemical parameters of grass carp. **(A)** Blood glucose; **(B)** blood total cholesterol; **(C)** blood triglycerides; **(D)** liver total cholesterol; **(E)** liver total triglycerides. Values are expressed as means ± SD. Differences were assessed using ANOVA and denoted as follows: ^∗^*p* < 0.05; ^∗∗^*p* < 0.01; NS, not significant.

Although the body weight and relative fatness (condition factor) were not significantly different between the BBR-fed and control group (Supplementary Figure S1), the levels of glucose and lipids in serum and liver were significantly affected by BBR in grass carp (Figure 1). The levels of glucose, TC, and TG in blood sera in the BBR-fed group were significantly (*p* < 0.05, *p* < 0.05 and *P* < 0.05, respectively) lower than those in the control group from days 3 to 9; however, these significant differences disappeared after day 14 when BBR-supplemented feeding stopped (Figure 1A–C). Compared with the control group, the levels of TC and TG in the liver were significantly higher (*p* < 0.05, *p* < 0.01, respectively) in the BBR group from day 7 to 21, but there were no significant differences days 1 to 3 before BBR-supplemented feeding, or on day 28 after BBR-supplemented feeding had stopped for 3 weeks (Figure 1D–E).

### Berberine Affected the Composition of Gut Microbiota

**FIGURE 2:**
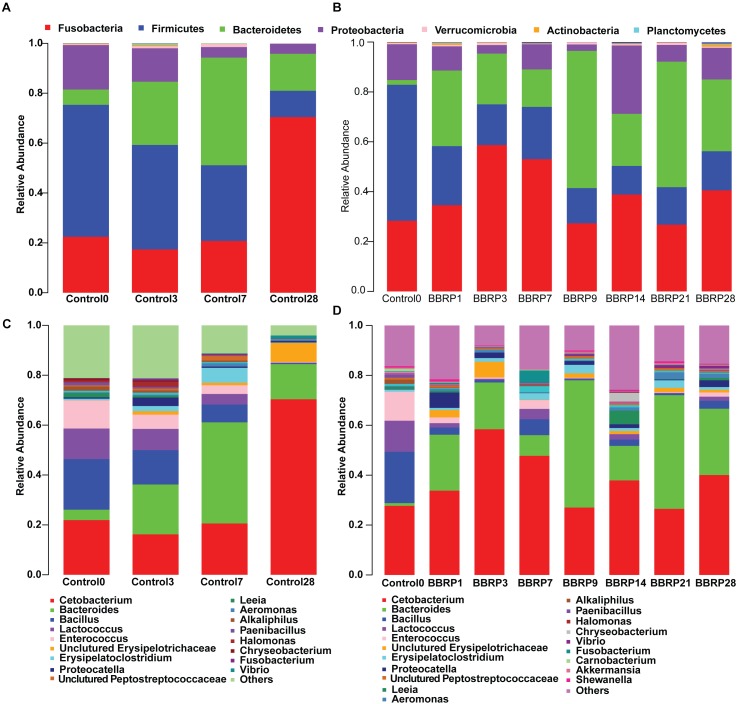
Average relative abundance of bacterial phyla and genera detected in gut microbiota of berberine-fed (BBR) and control grass carp. **(A)** Phyla structure in control group; **(B)** phyla structure in BBR-fed group; **(C)** genus structure in control group; **(D)** genus structure in BBR-fed group.

After trimming and filtering, a total of 4,142,382 high-quality reads were generated from 108 samples. More than 98% of the high-quality reads were retained and clustered to 1670 OTUs with 97% similarity after removing the ‘Unknown,’ “Cyanobacterial,” “chloroplast,” or “mitochondria” sequences. A total of 1561 OTUs were assigned to defined phyla using a RDP classifier with a bootstrap cutoff of 80%. The most abundant species of grass carp gut microbiota at the phylum level were Fusobacteria, Firmicutes, Bacteroidetes, and Proteobacteria (Figure 2A, more than 99% in total), while *Cetobacterium, Bacteroides, Bacillus, Lactococcus, Enterococcus, Erysipelatoclostridium*, and *Proteocatella* were dominant at the genus level (Figure 2C, more than 75% in total). The composition of gut microbiota changed dynamically as fish grew, both in the control and BBR-fed groups (Figure 2). For example, compared with day 0, the relative abundance of Fusobacteria, including *Cetobacterium*, increased significantly (from 25 to 75%), while the Firmicutes, including *Bacillus*, *Lactococcus*, and *Enterococcus* significantly decreased (from 55 to 10%) by day 28 in the control group. In addition, the relative abundance of *Bacteroides* was obviously increased from the beginning to the mid-sampling days, and significantly decreased at the end of the sampling days in the control group (from 5 to 40%, then to 15%). At the end of the sampling days, the relative abundance of potential pathogenic bacteria such as *Vibrio* was also significantly increased in the control group (Figure 2A,C).

The strong antimicrobial activity of BBR on both gram-positive and negative bacteria changed the pattern of gut microbiota, and the composition was significantly different in the BBR-fed group (Figure 2B,D) compared to the control group (Figure 2A,C) as the fish grew. Even though the diversity and relative abundance of some beneficial bacterium, such as *Bacteroides* were slightly decreased during BBR feeding (19.04, 12.77, and 6.24% on days 1, 3, and 7, respectively), the composition of gut microbiota was more diverse and stable after the BBR feeding was stopped than in the control group. For example, compared to the control group, the relative abundance of Firmicutes, Bacteroidetes, and Proteobacteria were significantly increased at day 28, while the abundant bacteria Fusobacteria were significantly decreased in the BBR group.

**FIGURE 3:**
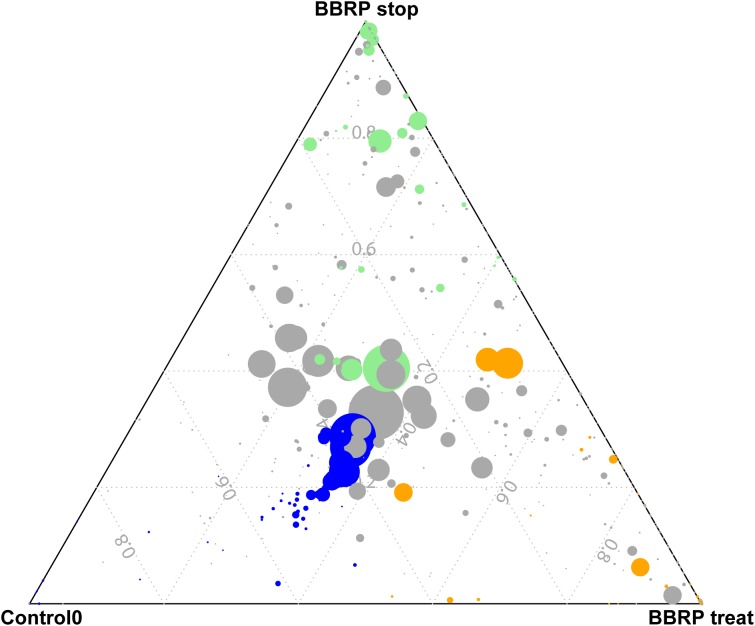
Specific enriched species at different growth stages (Control 0, during berberine (BBR) feeding and after BBR feeding was stopped) in BBR-fed group. Blue, enriched OTUs Control 0; Orange, enriched OTUs during BBR feeding; Green, enriched OTUs after BBR feeding stopped.

Even though most gut microbes were found in both groups, some species were enriched in different groups (Figure 3). In total, 51, 28, and 32 OTUs were enriched before BBR feeding, during BBR feeding, and after BBR feeding, respectively. The enriched OTUs before BBR feeding mainly belonged to Firmicutes, while most enriched OTUs during and after BBR feeding came from Proteobacteria, Planctomycetes, Bacteroidetes, and Firmicutes. When we compared the relative abundance of gut microbes on the same sampling day between the control and BBR group, we found that 48, 25, and 162 OTUs were significantly different on days 3, 7, and 28, respectively, further suggesting that BBR affected the composition of fish gut microbiota. The significantly enriched OTUs in the BBR group on day 28 were mainly Proteobacteria, Bacteroidetes, Firmicutes, and Actinobacteria. In addition, the ratio of Firmicutes to Bacteroidetes was significantly decreased as the fish grew during BBR feeding (from 8.33 to 1.45). Interestingly, this ratio significantly decreased from 8.33 to 0.25 between days 0 and 9 during BBR feeding, and increased from 0.25 to 1.45 from day 9 to 28 after BBR feeding stopped.

### Berberine Affected the Diversity of Gut Microbiota

**FIGURE 4:**
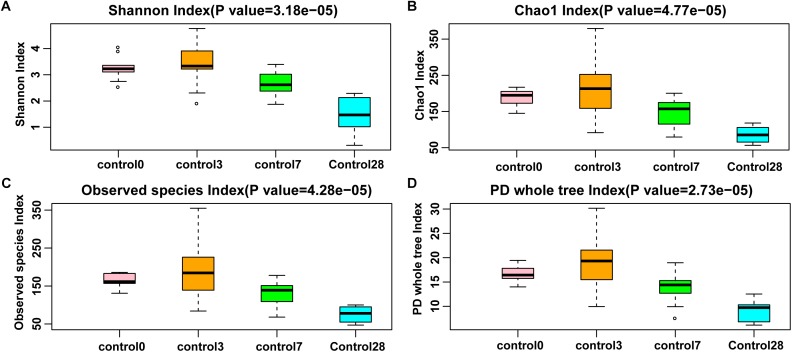
Species richness of control group of four alpha diversity indexes. **(A)** Shannon index; **(B)** Chao1 index; **(C)** Observed species index; and **(D)** Phylogenetic distance whole tree index. Significant differences were determined using the Kruskal-Wallis test.

All the rarefaction curves indicated that the sequencing depth was sufficient for each sample (Supplementary Figures S2,3). Compared with day 0, the alpha diversity on day 28 was significantly decreased in the control group (Kruskal-Wallis rank sum test, *p* < 0.0001, Figure 4). At the same time, the alpha diversity changed dynamically in the BBR feeding group (Kruskal-Wallis rank sum test, *p* < 0.05, Supplementary Figure S5) between days 0 and 28. The alpha diversity was significantly decreased during BBR feeding from days 3 to 7, and was significantly increased after BBR feeding stopped (from days 14 to 28). Compared to the control group, the alpha diversity was lower during BBR feeding, and became higher after BBR feeding stopped in the BBR group (Welch’s *t*-test, *p* < 0.05).

**FIGURE 5:**
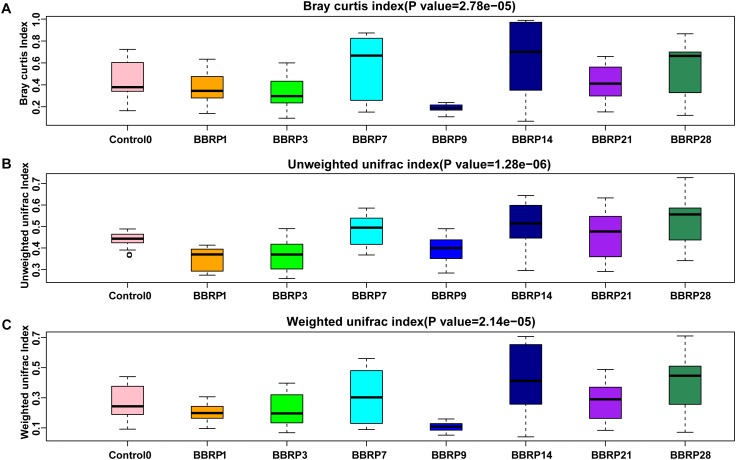
Sample distance within berberine (BBR) fed group on three beta-diversity metrics. **(A)** Bray Curtis; **(B)** Unweighted, and **(C)** weighted UniFrac distance index.

Compared with the control group, the gut microbial community distances were significant decreased from days 0 to 28 (Kruskal-Wallis rank sum test, *p* < 0.005, Supplementary Figure S4). In the BBR group, the distances between fish gut microbial communities within sample groups were significantly decreased during BBR feeding, and were significantly increased after BBR feeding stopped (Kruskal-Wallis rank sum test, *p* < 0.00001, Figure 5).

### Gut Microbiota Were Correlated With Biochemical Parameters of Serum and Liver

**FIGURE 6:**
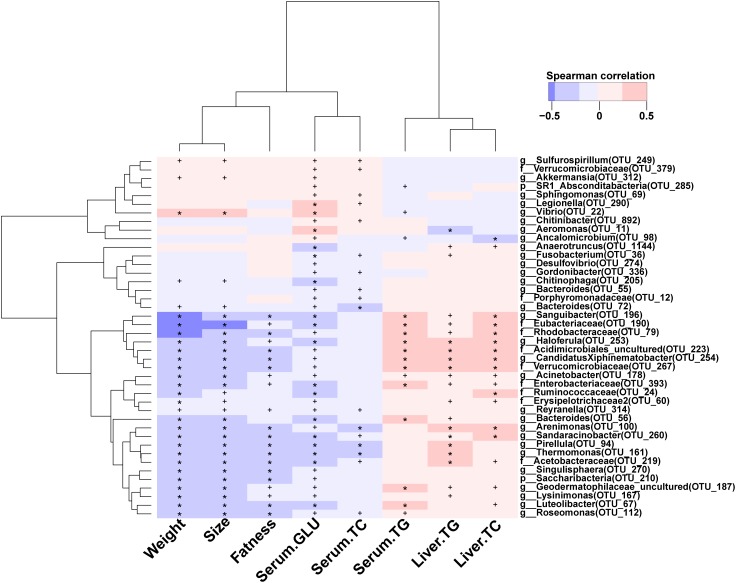
Correlation between host phenotype and gut bacterial species. ^∗^denotes *p* < 0.01; ^+^denotes *p* < 0.05.

The levels of glucose, TC, and TG in serum were significantly reduced during BBR feeding (Figure 1A–C). In total, 42 high abundance OTUs (relative abundance >0.01%) were significantly correlated with the levels of glucose, TC, and TG in serum and liver (Figure 6). Interestingly, 32 of 42 OTUs mainly distributed in Proteobacteria (9), Bacteroidetes (5), Actinobacteria (5), Verrucomicrobia (4), Firmicutes (3), Planctomycetes (3), Fusobacteria (1), Saccharibacteria (1), and Tenericutes (1) were significantly, negatively correlated with weight, body size, fatness, and the levels of glucose and TC in serum, and significantly, positively correlated with serum TG, liver total cholesterol and TG. Furthermore, most of the 32 OTUs were more abundant in the BBR group than the control group (Supplementary Figure S6), indicating these OTUs may have been affected by BBR. Besides the potential beneficial gut microbes, 10 of 42 OTUs were strongly, positively correlated with weight, body size, fatness, and the levels of glucose and TC in serum, and significantly, negatively correlated with the level of serum TG, liver TC, and TG.

## Discussion

Berberine can significantly reduce glucose levels in mammals (Leng et al., 2004; Yin et al., 2008; Zhang H. et al., 2010; Zhang et al., 2012; Xia et al., 2011; Dai et al., 2015; Wang Y. et al., 2017). The oral bioavailability of BBR is relatively low (Hua et al., 2007; Vuddanda et al., 2010; Chen et al., 2011). The maximum concentration (Cmax) of BBR in the plasma of rats is 4 ng/ml after oral administration of 100 mg/kg BBR (Liu et al., 2009; Zhang et al., 2012). The maximum concentration (Cmax) of BBR in the plasma of tilapia (*Oreochromis niloticus*) is 2.95 ng/mL after oral administration of 30 mg/kg BBR (Qin, 2014). Effective concentrations required for regulation genes or pathways in *in vitro* assays cannot be achieved as a result of low bioavailability of BBR (Zhang et al., 2012; Gu et al., 2015). Therefore, It is difficult to explain the mechanism of clinical effects of BBR based on systemic genes and pathways. However, it has been proved that the main action site of BBR is the gut (Sun et al., 2016). BBR directly impacts the gut microbiota of mice and causes lipid-lowering effects via sequential events (Sun et al., 2016; Tian et al., 2018). It has been also proved that the glucose-lowering effect of BBR in rats is associated with the shift of the gut microbiota structure in BBR-treated rats (Zhang et al., 2012). The dosage of BBR for anti-bacterial diseases in fish in the Chinese fisheries pharmacopeia is 15–30 mg/kg body weight (Yang, 2005). The present study is the first to report that oral administration of 30 mg/kg BBR, which is the Highest Permissible Dosage in the Chinese fisheries pharmacopeia, strongly reduced glucose level in grass carp. The mechanism by which BBR reduces glucose levels in grass carp was presumed to be the modulation of gut microbiota after oral administration. This was inferred from our results, as 32 high abundance OTUs (mainly in the BBR group) were significantly negatively correlated with levels of glucose (*p* < 0.05). These results broaden our view that BBR lowers blood glucose level in teleost fishes as well as mammals. In addition, BBR or BBR-derived products may be useful in the regulation of blood glucose levels and gut microbiota in grass carp culture.

Besides the effects on fish metabolism, BBR also dramatically affected the structure of host gut microbiota. Consistent with previous findings in mice (Zhang et al., 2012), we found that the structure and diversity of grass carp gut microbiota was significant affected by BBR, indicating its antimicrobial activity (Wu et al., 2012; Wang Y. et al., 2017). Interestingly, the diversity of grass carp gut microbiota was higher in the BBR group after it was stopped being fed BBR than in the control group, suggesting that BBR may maintain the stability and health of grass carp gut microbiota. Furthermore, due to the antimicrobial activity of BBR, some abundant bacteria, such as Firmicutes and Fusobacteria, were inhibited, releasing limited food and niche resources. Meanwhile, other gut microbes, such as Bacteroidetes, Proteobacteria, and Actinobacteria may have used these resources increasing the diversity of gut microbiota. Additionally, the ratio of Firmicutes and Bacteroidetes was significantly correlated with the fatness, TC and TG of serum in mammals (Ley et al., 2005, 2006; Eslinger et al., 2014; Hussain et al., 2016). Interestingly, we also found that the ratio of Firmicutes and Bacteroidetes was decreased as glucose, TC, and TG in serum decreased in the BBR group, indicating that BBR may affect the levels of serum glucose, TC, and TG by modifying the ratio of Firmicutes and Bacteroidetes. We also found that 10 and 32 abundant OTUs were significantly, positively and negatively correlated with serum glucose, respectively. Interestingly, some of the negatively correlated OTUs belonged to the potentially beneficial Bacteroidetes, while some of the positively correlated OTUs belonged to potential pathogens, such as *Vibrio*. Furthermore, most of the 32 negatively corelated OTUs were more abundant in the BBR group than in the control, indicating that these OTUs may have been affected by BBR and further affected the metabolism of grass carp.

## Conclusion

In summary, the levels of glucose, TC, and TG in blood were significantly decreased, and the diversity and structure of intestinal microbial bacteria in grass carp were affected by BBR-supplemented feed. BBR may have directly affected fish metabolism by controlling and modifying the structure of the gut microbiota, such as adjusting the ratio of Firmicutes to Bacteroidetes, increasing diversity, and recruiting more beneficial microbes. These findings indicate that BBR lowers blood glucose levels in teleost fishes via the gut microbiota, and that BBR or BBR-derived products may be used to maintain the growth and health of grass carp culture.

## Ethics Statement

The experiments protocols were approved by the animal Ethics Committee of the Guangdong Provincial Zoological Society, China (permit number GSZ-AW003).

## Author Contributions

HP, JX, and DL conceived and designed the experiments. HP, ZL, and CS performed the experiments with help from JX and DL. HP and HW collected the data. JX, HP, ZL, DL, and HW analyzed the data. HP, JX, DL, ZL, ZH, QZ, and DY co-wrote the manuscript. All authors discussed the results and commented on the manuscript.

## Conflict of Interest Statement

The authors declare that the research was conducted in the absence of any commercial or financial relationships that could be construed as a potential conflict of interest.
