# Supplementary material for: Berberine Influences Blood Glucose via Modulating the Gut Microbiome in Grass Carp
Source: Front Microbiol. 2019 May 9;10:1066. doi: 10.3389/fmicb.2019.01066 (PMC6520828; doi:10.3389/fmicb.2019.01066)
Supplement: Supplementary file 1 [file Data_Sheet_1.PDF]

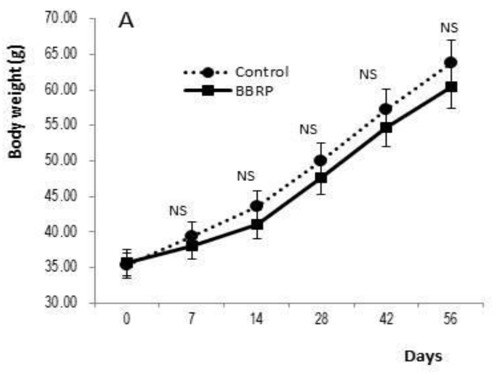


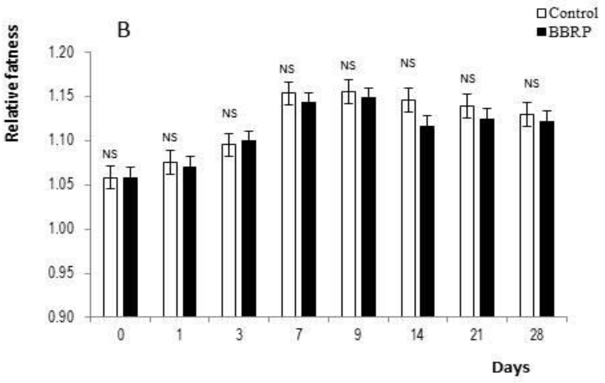


**Figure S1. Effects of berberine on body weight gain and the relative fatness of grass carp.** (A) Body weight gain of Control and BBRP group; (B) Relative fatness (RF) of Control and BBRP group. RF calculated are as follows: RF=100×body weight(g)/body length(cm) $^{3}$. Values were expressed as means ±SD. Differences were assessed by ANOVA and denoted as follows: $^{NS}$ not significant ($p>0.05$).

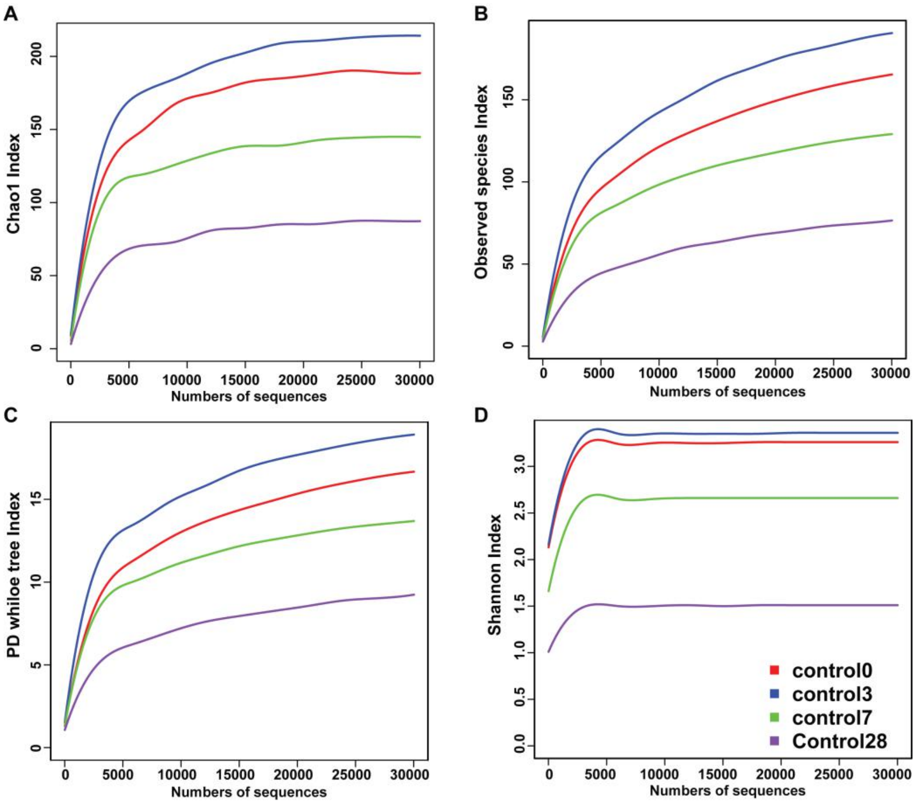


**Figure S2. Rarefaction curve of non-treatment samples based on four alpha diversity indexes.** (A) Chao 1 index; (B) Observed species index; (C) phylogenetic distance whole tree index; (D) Shannon Index.

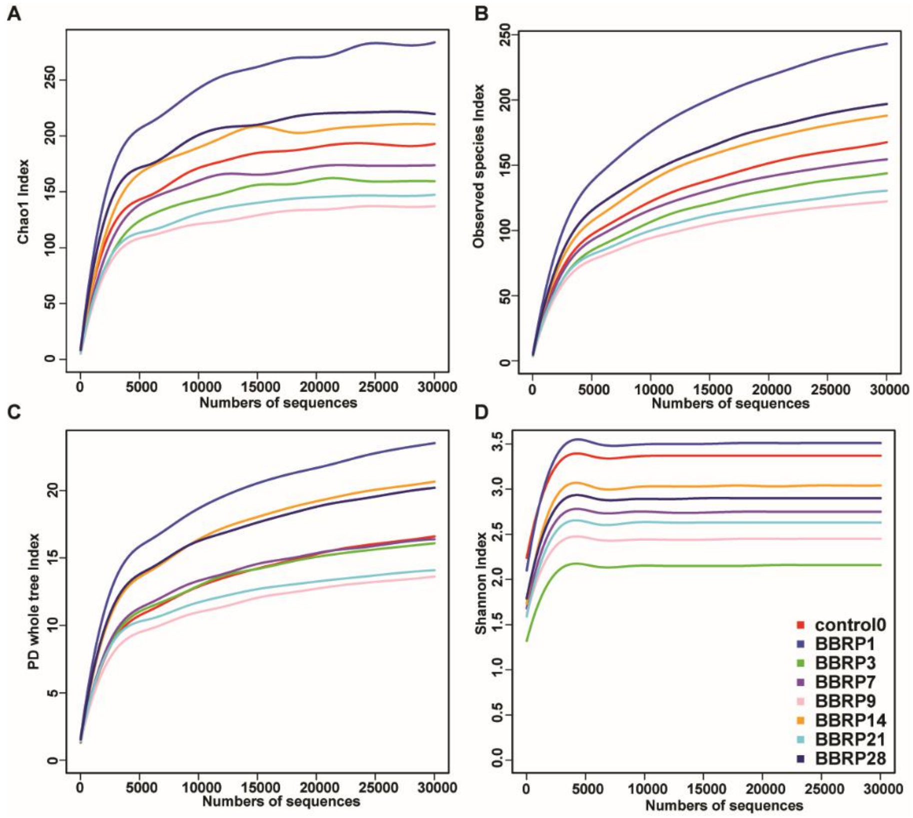


**Figure S3. Rarefaction curve of treatment samples based on four alpha diversity indexes.** (A) Chao1 index; (B) Observed species index; (C) phylogenetic distance whole tree index; (D) Shannon index.

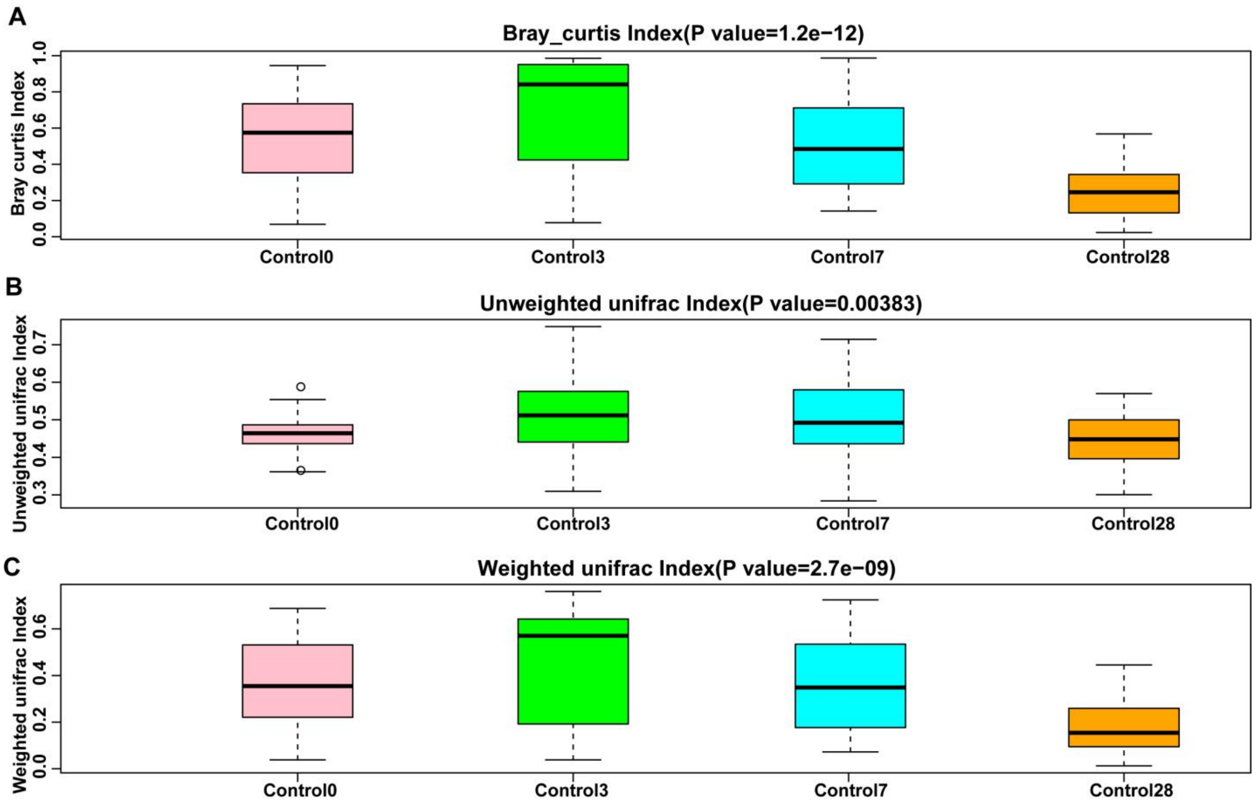


**Figure S4. Sample distance within group of non-treatment samples based on three beta-diversity indexes.** (A) Bray curtis index ; (B) Unweighted unifrac index; (C) weighted unifrac index.

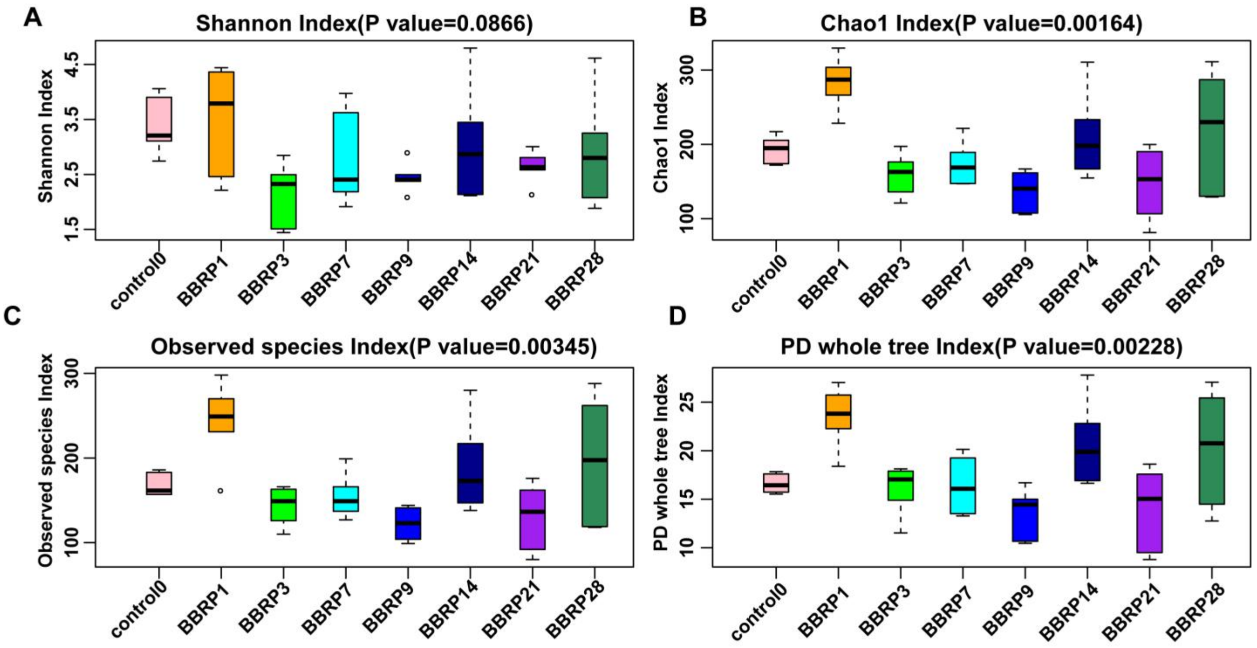


**Figure S5. Species richness of treatment samples based on four alpha diversity indexes.**

(A) Shannon index; (B) Chao1 index; (C) Observed species index; and (D) Phylogenetic distance whole tree index. The P value were calculated using Kruskal-Wallis test.

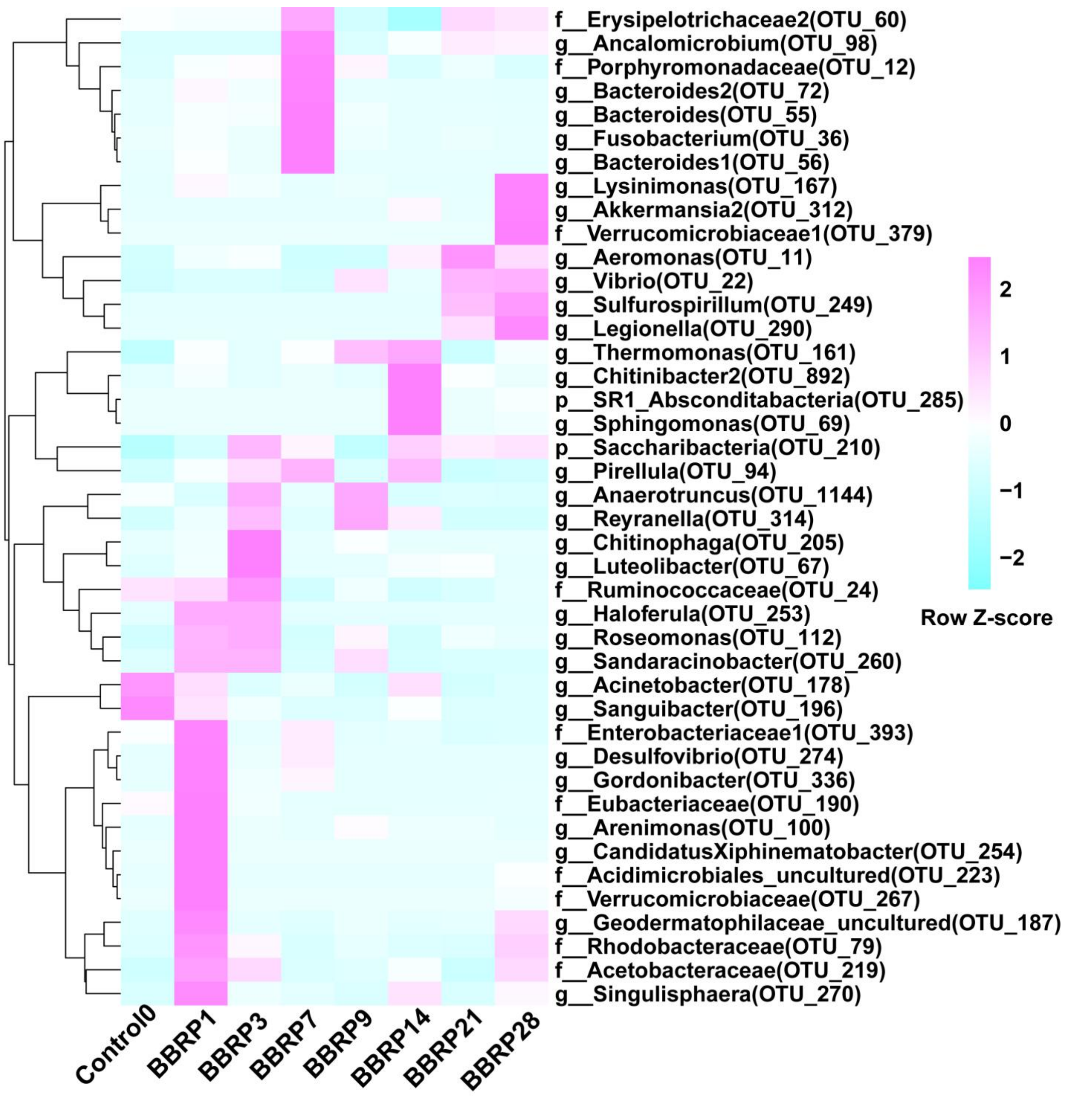


**Figure S6. The relative abundance of gut bacterial species which were significant related with the host phenotype.**
